# Supplementary material for: The Validity and Predictive Value of Blood-Based Biomarkers in Prediction of Response in the Treatment of Metastatic Non-Small Cell Lung Cancer: A Systematic Review
Source: Cancers (Basel). 2020 Apr 30;12(5):1120. doi: 10.3390/cancers12051120 (PMC7280996; doi:10.3390/cancers12051120)
Supplement: Supplementary file 1 [file cancers-12-01120-s001.zip › S1.docx]

S1: Search queries used in the systematic literature review.

Scopus:

(TITLE-ABS-KEY(nsclc OR "non*small cell" OR "non small cell") AND TITLE-ABS-KEY(*DNA OR *RNAs OR RNA OR exosom* OR genom* OR CTC OR CTCs OR "extracellular vesicl*") AND TITLE-ABS-KEY(blood OR "blood*based" OR serum)) AND PUBYEAR > 2013

PubMed

(((nsclc[Title/Abstract] OR "non*small cell"[Title/Abstract] OR "non small cell"[Title/Abstract])) AND (*DNA OR *RNAs OR RNA OR exosom* OR genom* OR CTC OR CTCs OR "extracellular vesicl*")) AND (blood OR "blood*based" OR serum) AND ( ( "2014/01/01"[PDat] : "2019/12/31"[PDat] ) )
